# Peer Interaction and Group Education for Adaptation to Disease in Adolescents with Type 1 Diabetes Mellitus

**DOI:** 10.12669/pjms.324.9809

**Published:** 2016

**Authors:** Sebahat Altundag, Meral Bayat

**Affiliations:** 1 Sebahat Altundag, PhD. Assistant Professor, Department of Pediatric Nursing, Pamukkale University Health Science Faculty, Denizli, Turkey; 2 Meral Bayat, Associate Professor, Department of Pediatric Nursing, Erciyes University Health Science Faculty, Kayseri, Turkey

**Keywords:** Adolescents, Adaptation, Nurse, Type 1 Diabetes, Social Interaction

## Abstract

**Objective:**

To evaluate the effects of group interaction and training in the adaptation process to disease in adolescents with type 1 diabetes mellitus (T1DM).

**Methods:**

This experimental study with pre- and post-test control groups was conducted in the pediatric endocrine clinic at a university hospital. The data were collected through descriptive data form, social support assessment scale, self-esteem inventory and information form. The data collection forms were administered four times with 3-month intervals to the study and control groups. Training, peer interaction and social support attempts were provided to the study group.

**Results:**

After the training and peer interaction, it was determined that there was a decrease in HbA1c levels and an increase in self-esteem (p<0.001) and mean scores of social support (p<0.05), and significant increase in knowledge levels (p<0.001). As for the adolescents in the control group, it was determined that there was no change in their HbA1c levels (p>0.05), and that there was no difference in their self-esteem scores, mean knowledge levels (p>0.05) in comparison with their pre-test scores.

**Conclusion:**

The training and peer interaction in adolescents were found to be effective in the adaptation process to the disease.

## INTRODUCTION

Type 1 diabetes mellitus (T1DM) is a common chronic disease in children and adolescents. Adolescents with T1DM are more likely to be engaged in mismanagement behaviors.^1^-^3^

Lifestyle changes due to intensive treatment programs and limitations in physical activities and daily life may restrict peer relationships and can lead to problems in peer interaction.^4^,^5^ Self-concept and self-esteem can be significantly affected by the disease in adolescents, they might be rejected by their peers, and they may not be able to establish good communication with their peers.^4^ Diabetes is a disease that requires lifelong compliance. The adolescent’s adaptation to the treatment can be facilitated with diabetes education, monitoring, counseling and support.^6^-^7^ Adolescents who gain independent decision-making skills and disease management ability through education show better adaptation and metabolic control.^8^ In addition, adolescents have been observed to be better in diabetes care of themselves when they had good communication with their peers.^9^

There are some descriptive studies on adolescents with T1DM focusing on training programs and peer support. However, due to the lack of an interventional study in our region on the effects of peer interaction on adolescents’ adaptation to disease, this study was considered necessary.

This study was conducted to determine the effect of interactive education with group interaction on adaptation to disease in adolescents with T1DM by using a control group experimental design with pre and posttests.

## METHODS

Ethical approval for this study was obtained from the ethics committee (25.01.2010/B.30.2.ERC.0.20./18) and the institution where the study was conducted. The researchers also explained the aims of the study to the adolescents with T1DM and their mothers and obtained their consent both verbally and in writing.

The study was conducted at a university hospital in the Aegean Region of Turkey using an experimental design with pre/post-tests and control group. Before the study, the outpatient records of the recent years were examined, and a total of 55 adolescents with T1DM between the ages of 12 and 14 years were determined. Forty of these adolescents who met the criteria for inclusion in the study (accepting to participate in the study, receiving quadruple insulin treatment, having no other chronic disorders, and having been diagnosed with T1DM for at least one year) were involved in the study. 20 of the adolescents were assigned to the study group and another 20 of them were assigned to the control group randomly. Two subjects were excluded from the study group since they could not be reached after the first interview. The study was completed with 38 adolescents (study group n=18, control group n=20).

When the post-power statistical analysis of the total pre/post-training scores that the T1DM adolescents in the study group obtained from the “Coopersmith Self-Esteem Scale” was carried out, the power, with alpha= 0.05, was calculated to be 1.00 respectively. With these results, the sample size was determined to be sufficient. The age, gender and income levels of the adolescents in the control and study groups were taken as matching criteria, and no significant difference was found between them (p>0.05).

The information form contains 14 questions regarding introductory information about the adolescents with T1DM. Children’s social support appraisal scale^10^ (cronbach’s alfa = 0.930) is made up of a total of 41 items. Maximum score that can be obtained from the scale is 205. In the study, Cronbach’s alpha was found to be 0.850 for the study group and 0.947 for the control group. Self-esteem inventory^11^ (cronbach’s alpha = 0.620, r = 0.65, r = 0.76) consists of 25 “yes-no” questions, and scores range from 0 to 100. In the study, the cronbach’s alpha values were found to be 0.885 for the study group and 0.954 for the control group. The diabetes mellitus knowledge test was created by the researchers in order to evaluate the adolescents’ diabetes training and measure the level of their general knowledge related to the disease. It is made up of twenty multiple choice questions. Maximum score that can be obtained from the test is 100, and a high score indicates a high level of knowledge. The opinions of four experts were sought when formulating the questions. Diabetes Education Guide for Adolescents (DEG) contains general information and advices about T1DM. The form of the guide was used after obtaining the opinions of four experts and making necessary adjustments.

The information form and scales were administered to the adolescents with T1DM in both study and control groups at the beginning of the study (the first month of the study).

### The Training stage of the study

The study group (n=18) was divided into 4 small groups of 4-5 subjects so that the training stage could be carried out interactively and effectively. The training of the groups, designated by drawing lots, took four sessions (each session took 35-45 minutes), and then the adolescents were given a training guide. There were breaks between the sessions for giving snacks to the adolescents. The training sessions started with small warm-up games, and then methods such as narrating, question-answer, demonstration, discussion and role-play were employed. One month after the training (the third month of the study), the scales were re-administered to the study and control groups.

### Social Adaptation and Consultation Stage

Social adaptation (peer interaction, interviews with family and counselors) and counseling activities were provided to the adolescents in the study group in order to monitor their peer interaction and adaptation to the disease. The study was also supported by dietitian, diabetes nursing, and child psychiatrist. Following the interactions (the sixth month of the study), the scales were re-administered to the study and control groups.

### Peer Interaction Stage

Peer interactions were performed in this period. To gain the experience of living with diabetes in social environments, activities such as going to the theatre and cinema, and then having lunch and a birthday party were performed.

### Post-test Stage

After the activities (the ninth month of the study), the scales were re-administered in the study and control groups.

During the study, the control group was given routine training and they answered the question about what they want to learn about T1DM. The Education Guide was also given to the control group at the end of the study.

### Data analysis

Statistical analysis was performed by using SPSS 15.0 for Windows software. The data were analyzed by using Chi-square test, student t test, variance analysis at repeated measurements and Cochran Q test. A value of p<0.05 was accepted as statistically significant.

## RESULTS

Based on the characteristics of the subjects, it was determined that most of the participants had had diabetes for 1-4 years (study group 66.7%, control group 55.0%), and that 61.1% of the study group adolescents went for checkups with the intervals of more than three months.

There was no statistically significant difference between baseline HbA1c levels of the study and control groups, while after the interventions, the HbA1c levels of the study group decreased, (p< 0.001) (Table-I). There was no change in the post-study HbA1c mean scores of the control group compared to their baseline scores (p = 0.993), the mean HbA1c levels of the study group were lower than those of the controls after the study, (p<0.05).

**Table-I:** Mean Hba1c Levels for the groups at measured times.*

HbA1c Value	Study Group M ± SD	Control Group M ± SD	T (p)
0 months	10.23 ± 2.39 ^a^	9.67 ± 2.02	-0.78 (.440)
3rd month	9.13 ± 2.47 ^b^	9.63 ± 1.95	0.69 (.494)
6th month	8.78 ± 1.69^b^	9.65 ± 2.59	1.23 (.227)
9th month	8.02 ± 1.66^b-c^	9.65 ± 2.13	2.62 (.013)
F *(p)*	14.43 (<.001)	0.00 (.993)	

* According to the results of the multiple comparison test, there was no difference between groups of the same superscript, but there was a difference between groups of different superscripts.

After the study, it was found that there was an increase in both mean self-respect scores of the adolescents in the trained, peer-interaction group (p<0.001), the self-respect score of the adolescents in the control group (p<0.005). In 0, 3, 6 months, there was no statistically significant difference between the self-respect mean scores of the study and control groups, but in 9^th^ month the difference between the two groups was found to be statistically significant (p<0.05) (Table-II).

**Table-II:** Mean Scores of Coopersmith Self-Esteem Scale for the groups at measured times.^*^

Coopersmith Self-Esteem	Study Group M ± SD	Control Group M ± SD	T (p)
0 months	65.55 ± 17.47 ^a^	67.80 ± 20.66 ^a-b^	-0.35 (.721)
3rd month	69.77 ± 16.76 ^c-a^	68.80 ± 19.96 ^a-b^	0.16 (.872)
6th month	77.33 ± 14.77 ^c^	66.60 ± 22.14 ^b^	1.73 (.091)
9th month	86.00 ± 10.01 ^b^	73.40 ± 19.69 ^a^	2.44 (.020)
F (p)	18.20 (< .001)	2.80 (.048)	

As for the adolescents in the study group, it was determined that, compared to 0 month, there was an increase in the mean scores of social support and in the mean scores for the support from friends and family subscales in 3^rd^, 6^th^ and 9^th^ months, and that the difference between them was statistically significant (p<0.05). Compared to 0 month, it was found in 3^rd^, 6^th^, and 9^th^ months for the control group adolescents that there was an increase in the mean scores for support from friends and teachers subscale scores and total scale scores (p<0.05).

It was determined that the post-study knowledge levels of the study group increased significantly compared to their baseline levels, (p<0.001), while the mean scores of the control group decreased (p>0.05) (Table-III).

**Table-III:** Mean Scores of Diabetes Information Test for the groups at measured times.^*^

Diabetes Information	Study Group M ± SD	Control Group M ± SD	T (p)
0 months	63.05 ± 15.91^a^	63.25 ± 15.91^a^	-0.03 (.970)
3rd month	66.11 ± 14.09^a^	60.75 ± 17.79^a^	1.02 (.314)
6th month	76.05 ± 11.93^b^	60.25 ± 16.58^a^	3.33 (.002)
9th month	95.55 ± 5.39^c^	61.75 ± 19.75^a^	7.02 (.001)
F (p)	46.93 (< .001)	0.61 (.510)	

## DISCUSSION

In the study, significant changes were observed in the HbA1c levels of the study group; however there were no significant changes in the control group. Similar to our study, it has been shown in various other studies that planned training given to individuals diagnosed with DM led to a reduction in HbA1c values.^12^-^15^

Having a disease seriously affects the self-concept and self-esteem in adolescence, while an individual is trying to develop his/her identity. And a tendency towards introversion can be observed.^6^ It is important to preserve self-esteem in T1DM.^6^ Self-esteem should be high in children who find themselves worthy and can cope with their chronic disease.^6^ While there was no difference between the self-esteem mean scores of the study and control groups in the first month, the level of their self-esteem was determined to increase at the end of the study. Studies have found that adolescents with T1DM who manage diabetes well have high self-esteem.^16^ Despite the negative effects of chronic disease on self-esteem,^4^-^6^ as a result of the training and group-interaction meetings held in our study, the self-esteem of the study group was found to be high. This finding was thought to stem from training after diagnosis, varied social activities with peers, expressing emotions and support from friends.

It was determined in the study that the social support scores of adolescents in both groups increased. However, the increase in the study group was observed to be higher. This result was thought to indicate that the trainings and peer interaction meetings in the study group were effective, and that the repetition of the questionnaire items in the control group created a stimulus in this regard. Social support enables adolescents with T1DM, a disorder which causes physical, emotional and psychological changes, to cope with difficulties associated with the management and treatment of the.^17^ This support is necessary for the prevention and diagnosis of psychological problems, adolescents’ adaptation to the, control of DM, and for a good diabetes management.^18^,^19^ In related studies, it has been indicated that adolescents with T1DM are supported on different issues by family and friends.^12^,^18^,^19^ Social support is important in enabling adolescents to pass through this life-stage more healthily, and when it is withdrawn, the adolescents’ self-assessment of health worsens.^20^ Social support for children is provided by parents and family, while it is provided by peer groups and teachers for adolescents.^17^ Activating social support systems and increasing support groups in adolescents was considered to be influential in diabetes management and helping adapt to the disease.

Diabetes training is accepted to be a basis for glycemic control and diabetes management. Training programs both increase knowledge of diabetes mellitus and make the individual more comfortable psychologically.^14^ Because of the abstract ideas developed in adolescence period, it can be beneficial to explain and the treatment program with the help of examples and pictures. Moreover, support groups can be used as a good instrument for training.^21^ It has been found in studies which provided planned diabetes training that the knowledge levels in the training groups increased significantly, and that HbA1c levels fell.^21^-^24^ It was determined that the knowledge level of the study group adolescents increased, while there was no significant difference in that of the control group adolescents. It was thought that the formation of training content according to adolescents’ needs and an interactive training in an appropriate atmosphere for similar needs and requirements, and the adolescents’ collaboration in sharing opinions and ideas were effective in increasing knowledge levels of the study group.

In the study, the adolescents with T1DM in the study group were involved in training, peer interaction, and social support activities, and it was determined at the end of the study that there was a decrease in HbA1c level and increase in social support and self-esteem levels. Activities such as giving constant and regular training and providing peer group interaction to adolescents with T1DM, and creating social support groups for them guided by nurses and team members are considered to be important in terms of adaptation to the disease.
